# Complementary and Alternative Medicine use in women during pregnancy: do their healthcare providers know?

**DOI:** 10.1186/1472-6882-14-85

**Published:** 2014-03-04

**Authors:** Lisa Strouss, Amy Mackley, Ursula Guillen, David A Paul, Robert Locke

**Affiliations:** 1 Division of Neonatology, Department of Pediatrics, Christiana Care Health System, 4745 Ogletown-Stanton Road, Newark, DE, USA; 2 Jefferson Medical College, 1025 Walnut Street, Philadelphia, PA, USA

**Keywords:** Complementary and Alternative Medicine, Pregnancy, Maternal-fetal health, Patient-physician communication, Self-care, Prevalence, Cross-sectional study

## Abstract

**Background:**

The National Institutes of Health reported in 2007 that approximately 38% of United States adults have used at least one type of Complementary and Alternative Medicine (CAM). There are no studies available that assess general CAM use in US pregnant women.

The objectives of our study were to determine the prevalence and type of CAM use during pregnancy at one medical center; understand who is using CAM and why they are using it; and assess the state of patients’ CAM use disclosure to their obstetrical providers.

**Methods:**

A cross-sectional survey study of post-partum women was done to assess self-reported CAM use during pregnancy. Results of this survey were compared to results from a previous survey performed by this research team in 2006. Data were analyzed using binary logistic regression.

**Results:**

In 2013, 153 women completed the survey, yielding a response rate of 74.3%. Seventy-two percent and 68.5% of participants reported CAM use during their pregnancies in 2006 and 2013 respectively. The percentage of participants who reported discussing CAM use with their obstetrical providers was less than 1% in 2006 and 50% in 2013. Increased use of different CAM therapies was associated with increased maternal age, primagravida, being US-born, and having a college education (p ≤ 0.05). However, these factors were poor predictors of CAM use.

**Conclusions:**

Given the frequency of CAM use and the difficulty in predicting who is using it, obstetrical providers should consider being informed about CAM and incorporating discussions about its use into routine patient assessments.

## Background

In 2007, approximately 38 percent of United States adults (83 million people) used at least one type of Complementary and Alternative Medicine (CAM) [1]. In that year alone, they spent $33.9 billion out-of-pocket on CAM therapies [2]. However, little is known about CAM use in US pregnant women. This lack of knowledge poses a unique challenge to women and health care providers [3]. During their pregnancies, many women are concerned about the potentially harmful effects of conventional medicine on their babies [4] but, because CAM is often thought of as natural and without risk [5,6], women frequently are not aware of the possible negative effects of their CAM use [7].

Complementary and Alternative Medicine is described by the National Institutes of Health’s National Center for Complementary and Alternative Medicine as “the array of health care approaches with a history of use or origins outside of mainstream medicine” [8]. It encompasses a wide range of therapies that can be divided into five categories: Alternative Medicine practices (traditional Chinese medicine, acupuncture, homeopathy, etc.), Mind-body interventions (meditation, yoga, imagery, prayer, etc.), Biologic-based therapies (herbal medicine or teas, dietary supplements, probiotics, etc.), Manipulative and Body-based methods (osteopathic and chiropractic manipulation, massage therapy, etc.), and Energy Healing therapies (qigong, tai chi, Reiki, etc.). These therapies can be used either alongside conventional medicine (Complementary Medicine) or by themselves (Alternative Medicine).

With such a wide array of therapies considered to be CAM, it is important to note that different types and categories of CAM have different risk profiles and can have a wide range of implications for the health of the mother and fetus. For example, no harmful effects have been linked to Mind-body CAM interventions, such as yoga, hypnotherapy, and imagery [9], and it would be unlikely to encounter an adverse effect from prayer or energy healing when used in concert with conventional medicine. However, for Alternative Medicine practices, Biologic-based therapies, and Manipulative and Body-based methods, the risk versus benefit calculation is less straightforward. The use of Biologic-based therapies, such as raspberry tea leaf and some herbal and vitamin supplements, during pregnancy has been associated with adverse events, such as premature closure of the ductus arteriosus and neonatal hyponatremic seizures [10], while the use of other supplements, such as prenatal vitamins, has been widely accepted by the field. Some evidence of the benefit of certain types of Alternative Medicine practices and Manipulative methods, such as accustimulation for pregnancy-induced nausea [11] and osteopathic manipulation for back pain during the third trimester of pregnancy [12], have also been established.

In studies of CAM use from countries outside the US, 50-70% of pregnant women studied were found to use at least one type of CAM [7,13-15]. A UK study found that despite these high levels of use, most obstetrical providers did not ask about CAM use during pregnancy, and one-third of mothers did not disclose their CAM use to their obstetrical providers [15]. A variety of predictors of maternal CAM use have been found, including higher education, ethnicity, and income in Germany [7], parity, higher education, and CAM use before pregnancy in the UK [15], and higher education and Caucasian origin in Switzerland [14]. The most common types of CAM used during pregnancy varied amongst the countries [16].

Our study’s objectives were 1) to learn about CAM use during pregnancy in a single U.S medical center; 2) to look for changes in CAM use between 2006 and 2013; 3) to analyze maternal factors associated with CAM use; and 4) to understand the state of CAM use disclosure between obstetrical provider and patient. We hypothesized that, in our population, CAM use would be high; it would be associated with higher education and income; and the quality of communication would be low between patient and provider. We also hypothesized that CAM use would increase from 2006 to 2013.

## Methods

A cross-sectional survey study was done at two time points. The first survey results were collected during a four month enrollment period in 2006; the second set of results was collected during a two month enrollment period in 2013. The sample population for the 2013 survey included women who had given birth to infants cared for on the Well Baby Newborn Unit (WBN) or in the Neonatal Intensive Care Unit (NICU) at Christiana Hospital, a tertiary care center on the East Coast. The 2006 survey was only given to women on the WBN. Both of the sample populations were convenience samples. Women with infants in the WBN were surveyed during their post-partum hospital stays, while women with infants in the NICU were surveyed during their infants’ hospital stays. Women less than 18 years of age, women of limited English proficiency, and post-partum women whose infants died within 24 hours of delivery were excluded from the study. Approval was attained from the Christiana Care Institutional Review Board.

The survey was designed and validated by six physicians, nurses, and mothers. There was no previously validated survey available. The survey consisted of five tables with questions about 22 different kinds of CAM therapies. For each therapy, there were four questions, asking whether CAM had been used during this recent pregnancy, the frequency of its use, and the obstetrical provider’s knowledge of and response to its use. The survey also contained 11 Likert scale questions from “Strongly Disagree” (5) to “Strongly Agree” (1), which asked about reasons for and attitudes toward CAM use. Participants were asked about prior CAM use, the use of other substances during pregnancy, who recommended CAM use, and the perceived benefit or harm of CAM. Demographic information was also collected. There were 48 questions in total. After providing informed consent, participants were asked to complete the survey and place it in a sealed envelope, which was later collected.

Survey responses were analyzed using SPSS (Version 20.0). We found differences in the breakdown of race and ethnicity between our sample population and the general population of women at Christiana Hospital. The data were weight-adjusted accordingly. Demographic information and CAM use data between the 2006 and 2013 groups, as well as between the 2013 NICU and WBN groups, were analyzed using Chi square test. With no significant differences in demographics and CAM use between the 2013 NICU and WBN groups, we combined them into one 2013 group for comparison with the 2006 group. Although the populations for the two time periods were different—the 2013 study looked at both NICU and WBN women and the 2006 study looked only at WBN women—and there were some demographic differences between the two groups, they were determined to be similar enough for comparison.

For the 2013 group and a combination of both the 2006 and 2013 groups, we used Mann–Whitney, Chi Square, and Binary Logistic Regression to analyze CAM use by maternal factors including age, race/ethnicity, country of origin, previous pregnancy, use of a midwife, public insurance, employment outside of the home, marital status, any college education, and mode of delivery. We looked at these factors by dividing CAM use into three groups to see if different categories of CAM therapies had users with different characteristics. The three categories we used were CAM use, CAM use excluding prayer, and CAM use excluding prayer, special diet, and supplements. Prayer was separated from the rest of the CAM therapies because people could consider prayer to be more religious than medical. Prayer, supplements, and special diets were separated because they were the more “mainstream” CAM therapies in our study. Finally, we compared the frequency and type of CAM use, provider response to CAM use, and Likert scale responses between the 2006 and 2013 data. P values less than or equal to 0.05 were considered statistically significant.

## Results

In 2013, 206 women (142 WBN, 64 NICU) were approached for this study, and a total of 153 (102 WBN, 51 NICU) were enrolled and completed the survey for a response rate of 74.3% (71.8% WBN, 79.7% NICU). In 2006, 201 out of 292 post-partum women (all WBN) were enrolled and completed the survey for a response rate of 69%. Table 1 contains the demographic information for the 2006 and 2013 samples, as well as information on the NICU and WBN groups within the 2013 sample. Median gravity and parity, race/ethnicity, insurance type, and type of obstetrical provider were different between 2006 and 2013 participants (p < 0.05). There were no significant statistical differences between 2013 NICU and WBN participants.

**Table 1:** Demographics of the 2006 and 2013 (NICU and WBN) Participants

**Demographic variables**	**2006 vs. 2013**			**2013**		
	**2006**	**2013**	**p-value**	**NICU**	**Well baby**	**p-value**
Total number of participants	201	153		51	102	
Mean age of mother (yrs)	29 ± 5	29 ± 5	0.90	28 ± 5	31 ± 5%	0.78
Median gravidity	2	2	0.03	2	2	0.61
Median parity	1	2	0.01	2	2	0.10
Mean gestational age (wks)	38 ± 1	37 ± 5	0.80	33 ± 6	39 ± 1	0.25
Delivery type						
Vaginal	60%	60%	0.92	48%	64%	0.06
Sex of infant						
Female	50%	52%	0.71	54%	54%	0.99
Marital status						
Married	72%	57%	0.31	46%	62%	0.07
Race/Ethnicity						
White, Non-Hispanic	68%	56%	0.02	50%	59%	0.30
Work						
Full-time	58%	64%	0.25	67%	62%	0.55
Education level						
College educated	43%	53%	0.06	47%	55%	0.33
Insurance						
Public	22%	36%	0.01	44%	31%	0.11
Where mom born						
US-born	92%	88%	0.22	92%	87%	0.32
Obstetrical provider						
Mid-wife	4%	14%	0.01	19%	11%	0.15

Comparing the 2006 and 2013 participants, median gravity and parity, race/ethnicity, insurance type, and obstetrical provider were all significantly different (p < 0.05). No statistically significant differences were found between the 2013 NICU and WBN groups.

### CAM use

**Figure 1:**
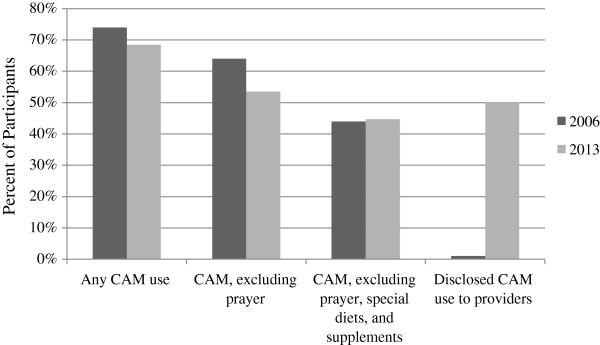
**Frequency and disclosure of CAM use in 2006 and 2013.** CAM use excluding prayer was significantly more frequent in 2006 than 2013. The percent of participants who disclosed their non-dietary CAM use to providers increased from 1% in 2006 to 50% in 2013 (p < 0.05).

**Figure 2:**
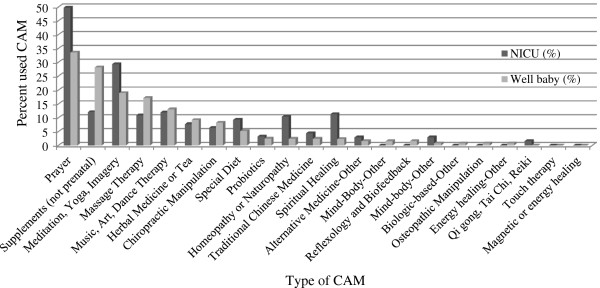
**Percent of NICU and WBN participants who used each type of CAM therapy in 2013.** Homeopathy/naturopathy (p = 0.032), spiritual healing (p = 0.011), and prayer (p = 0.05) were all statistically more likely to be used in the NICU than in the WBN participants.

CAM use was analyzed by year and by unit (NICU and WBN). In both 2006 and 2013, almost two-thirds of all participants used at least one type of CAM. Figure 1 demonstrates the percent of participants who used CAM in 2006 and 2013 according to the different categories of CAM therapies. The top nine most common CAM therapies used in both 2006 and 2013 were prayer, supplements, massage therapy, meditation, yoga, teas, music therapy, chiropractor, and special diet. Figure 2 separates the types of CAM therapies used by the 2013 NICU and WBN participants. Homeopathy/naturopathy (p = 0.032), spiritual healing (p = 0.011), and prayer (p = 0.05) were all more likely to be used in the NICU than in the WBN participants (Figure 2). Supplements (p = 0.087) approached statistical significance with more use in the WBN than in NICU. Participants were also asked how frequently they used medications and substances other than CAM (Table 2). Prescription medications were used less in 2013 than in 2006 (p < 0.05).

**Table 2:** Participants’ use of medications and substances other than CAM

	**2013**	**2006**	**p-value**
Over the counter medicine	78.1%	84.0%	0.15
Prescription medicine	51.0%	68.0%	0.04
Cigarettes	9.3%	13.0%	0.23
Caffeine	75.5%	80.0%	0.37

Prescription medications were used less in 2013 than in 2006 (p < .05).

### Maternal factors associated with CAM use

Table 3 shows maternal factors associated with different categories of CAM use for 2013 alone, as well as for 2006 and 2013 together. In multivariate analysis of the combined 2006 and 2013 data, any college education and a vaginal delivery were associated with higher rates of CAM use. The overall predictability of the model remained low, limiting its clinical usefulness.

**Table 3:** Prediction of CAM use - multivariate analysis

**Category of CAM use**	**Year - 2013**	**OR**	**95% CI**
**Any CAM**	Maternal age	1.11	1.04 – 1.18
**CAM excluding prayer**	College education	2.98	1.23 – 7.21
	Maternal age	1.10	1.019 – 1.20
	US born	5.30	1.45 – 19.41
	Employed outside home	0.30	0.13 – 0.71
	Previous pregnancy	0.21	0.08 – 0.51
**CAM excluding prayer, special diet, and supplements**	College education	3.33	1.60 – 5.52
	US born	3.89	1.06 – 14.32
	Midwife	2.89	1.05 – 7.94
	**2006 & 2013 combined data**	**OR**	**95% CI**
**Any CAM**	College education	3.31	2.04 – 5.37
	Vaginal delivery	2.02	1.21 - 3.37
**CAM excluding prayer**	College education	3.05	1.94 – 4.79
	Year 2013	0.61	0.39 – 0.95
	Vaginal delivery	1.56	0.99 - 2.58
**CAM excluding prayer, dietary supplements**	College education	2.76	1.75 – 4.37
	Vaginal delivery	1.53	0.98 - 2.38

Although models were statistically significant, the ability to accurately predict CAM use or non-use was clinically poor (61 -73%). Maternal factors evaluated were as follows: maternal age, race/ethnicity, US-born, previous pregnancy, use of a midwife, public insurance, employed outside of the home, marital status, any college education and mode of delivery.

### Maternal attitudes toward CAM use

Table 4 displays the Likert scale results of the participants’ responses to statements regarding CAM in both 2006 and 2013. During both time periods, the majority of CAM users was happy with traditional medical care, had used CAM prior to pregnancy, and used CAM to improve their pregnancy health and experience and/or to benefit their baby. The only response that differed between the two years was the percent of participants who used CAM during pregnancy because of unhappiness with conventional medicine (1% in 2006 and 10.3% in 2013, p <0.05). Most participants (80.3%) did not consider the CAM they used to be part of their culture. In non-Likert scale questions that asked about benefit and harm, 96.1% of participants thought that at least some of the CAM they used was beneficial, and only 1.3% of participants thought that any of the CAM they used was harmful. One person reported chiropractic manipulation as harmful, and one person reported massage as harmful.

**Table 4:** Maternal attitudes toward CAM use - 2006 vs. 2013

**Question about CAM use**	**Strongly agree/agree**		**Strongly disagree/disagree**		**No opinion**	
	**2013**	**2006**	**2013**	**2006**	**2013**	**2006**
**Reasons for CAM use:**						
Used CAM previously	60.1%	66.0%	16.5%	14.0%	23.4%	21.0%
Felt would improve health and experience	74.2%	73.0%	7.6%	7.0%	18.2%	20.0%
Felt would be beneficial to baby	69.3%	63.0%	10.0%	8.0%	20.7%	28.0%
Unhappy with traditional western medical care	10.3%	1.0%	69.2%	80.0%	21.4%	18.0%
Comfortable/happy using traditional western medicine alone during pregnancy and did not ask about or use CAM therapy	40.0%	*	27.0%	*	32.9%	*
Chose not to use CAM because did not know about CAM treatments	40.6%	*	39.7%	*	19.6%	*
**Obstetrical provider and CAM use:**						
Comfortable asking Obstetrical provider about CAM used during pregnancy	64.6%	72.0%	11.8%	4.0%	23.6%	24.0%
Comfortable informing Obstetrical Provider about CAM used during pregnancy	69.6%	78.0%	9.7%	7.0%	20.7%	24.0%
Chose not to use CAM during this pregnancy because uncomfortable asking obstetrical care team	3.6%	*	67.2%	*	29.3%	*
**Perception of the benefit and harm of CAM:**						
CAM used during pregnancy was beneficial/helpful	79.8%	89.0%	5.5%	1.0%	14.7%	10.0%
CAM used during pregnancy was harmful	4.3%	5.0%	80.3%	86.0%	15.4%	9.0%

Only CAM used because the participants “were unhappy with traditional western medical care” was significantly different between the two years (p < 0.05).

*Data was not collected in 2006.

### The obstetrical provider and CAM use

**Figure 3:**
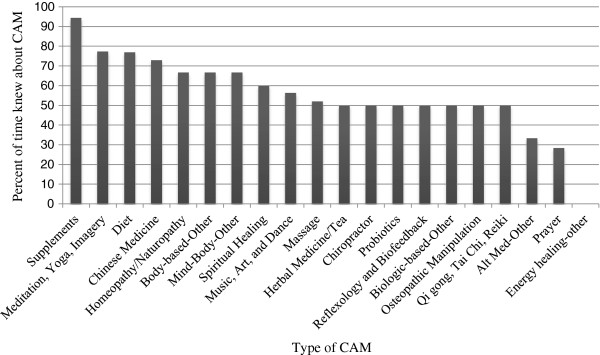
**Obstetrical provider knowledge of CAM use in 2013.** Women reported that they disclosed supplement use to their obstetrical providers most frequently (94.4%). Prayer use was one of the CAM therapies of which providers were least likely to be aware (28.4%). The providers’ response to CAM use disclosure was perceived by women as positive or neutral over 98% of the time.

In 2013, participants reported that their obstetrical providers knew about their CAM use 60.8% of the time. The percent of participants who disclosed their non-dietary CAM use to providers increased from 1% in 2006 to 50% in 2013 (p < 0.05) (Figure 1). Table 4 contains information about the participants’ level of comfort with talking to their obstetrical providers about CAM use in both 2006 and 2013. Only 1.6% of participants perceived their providers’ responses to CAM use disclosure as negative. There was no difference in perceived provider response between the NICU and WBN participants (negative responses: p = 0.164, positive responses: p = 0.362, neutral responses: p = 0.691). Participants reported that their obstetrical providers knew about supplement use most frequently (94.4%) and prayer use least frequently (28.4%) (Figure 3). Family and friends made up 74.7% of CAM recommenders while Obstetrical Providers made up 15.5% of CAM recommenders.

## Discussion

In a single tertiary care center on the East Coast of the United States, Complementary and Alternative Medicine is used commonly by women during their pregnancies, with 68.5% of women surveyed in 2013 reporting that they used at least one type of CAM. This prevalence closely matches those reported in the UK (57.1%), Switzerland (69%), Australia (50%), and Germany (50.7%) [7,13-15]. In spite of similarities in prevalence of CAM use, the types of therapies used by women in each country vary greatly [16]. In 2013, the top five most common types of CAM therapies used in our population were prayer; supplements; yoga, meditation, and imagery; massage; and music, art, and dance therapy. These are similar to the top four CAM therapies used in the UK (vitamins, massage, yoga, and relaxation) [15], but fairly different from the top three CAM therapies reported in Germany (homeopathy, acupuncture, and massage) [7]. Just as overall rates of CAM use in our population stayed around two-thirds between 2006 and 2013, the therapies participants used also remained very similar. Three of the top five CAM therapies used in both years were Mind-body interventions with low potential for adverse effects.

We also found minimal differences in the frequency and type of CAM used between NICU and WBN participants. One significant difference was the higher use of spiritual healing, prayer, and homeopathy/naturopathy in NICU participants. There is evidence to suggest that people experiencing certain health problems are more likely to use CAM [17]. This could also hold true for women when it becomes apparent during pregnancy that their infant may have to spend time in the NICU. Unfortunately we were not able to separate the NICU participants who knew about complications beforehand from the participants whose infants had an unexpected NICU admission.

CAM use in pregnant women is associated with primiparous older women of higher education and income who have physical health problems and previous complementary medicine use [16]. When we analyzed these variables, as well as other factors, we found multiple statistically significant relationships. However, it is important to note that these statistically significant factors are not good enough predictors of CAM use to be useful clinically. This inability to accurately predict CAM use by these demographic variables emphasizes that “consumers of complementary and alternative products or services are far from a homogeneous group with similar beliefs, motivations, and needs” [16]. Because the group of women who use CAM is so diverse, communication about CAM becomes all the more important. Obstetrical providers cannot rely on demographic information to accurately predict who is using CAM; instead, they must specifically ask their patients about it if they want reliable information.

Although it is difficult to predict which patients are using CAM therapies, common trends help explain why CAM is used. In our study population, most participants who used CAM made that decision because they “felt it would improve their health and experience” or because they “felt it would be beneficial to their baby.” This evidence supports the concept of a “risk society [18],” in which growing CAM use could be a sign of “a desire for personal fulfillment and need for autonomy and active participation in healthcare during pregnancy and childbirth” [19]. CAM could provide women with an opportunity, often thought of as risk free, to have a positive effect on both themselves and their infants. However, the desire to use CAM is not just limited to pregnancy. The majority of participants said they used CAM during their pregnancies because they had used it previously. Finally, a relatively small number of participants, 1% in 2006 and 10% in 2013, stated they used CAM because they were unhappy with conventional medicine. This shows providers that CAM use should not be seen as an expression of dissatisfaction, but rather may be something that patients value along with conventional medicine [20]. Even so, because there was a rise in dissatisfaction between the two time periods, investigating explanations for this increase would be a good area for future research.

In the 2013 study, we found that the obstetrical provider knew about CAM use 60.8% of the time. This is similar to the rates found in the UK [15]. It is also comparable to the 20-77% of US cancer patients who disclosed their CAM use to doctors [21], and to the two-thirds of US Hispanic women who disclosed information about supplement use to their physicians [22]. Our study shows that there has been a substantial increase in the reporting of CAM use to obstetrical providers between 2006 and 2013, despite a lack of reported change in the participants’ comfort asking or providing information about CAM. This increase in provider knowledge between the two time periods could be the result of providers becoming more aware of how common CAM use is. Seven years ago, providers may not have directly asked questions about CAM use; and without the provider asking about it specifically, the patient may have never thought to disclose it. Nevertheless, even with this remarkable improvement in CAM use disclosure over the seven year time gap, it is important to recognize that half of the participants’ CAM use still is not known by their providers.

There were significant variations in the likelihood that participants would share their CAM use with providers. Supplements, the second most commonly used CAM therapy, were most likely to be disclosed, while prayer, the most common CAM therapy, was one of the least likely to be shared. This may reflect the perception that supplements are well-received by the medical field, which may make patients more comfortable and willing to share the information with their providers. This trend is a positive finding since some supplements have been associated with adverse effects [10]. Conversely, prayer, which has not been associated with negative health effects, is often associated with a person’s religious beliefs and may not be perceived as something that needs to be discussed with providers. Nonetheless, having knowledge about the practices and beliefs of the mother and family is still valuable, especially in the event of complications for the mother or infant.

When a conversation about CAM use does take place, participants perceived their obstetrical providers’ responses as largely positive. The two reported perceived negative responses were toward osteopathic manipulation and qigong, tai chi, and reiki. Since we did not ask the corresponding obstetricians about their attitudes toward CAM, it is impossible to assess whether this accurately reflects the provider’s actual attitude. In Australian and Israeli studies where obstetricians were asked directly about their attitudes toward CAM use, they too found favorable responses from providers [23,24]. As was discussed previously, different types of CAM therapies have a wide range of risk profiles. Assuming that the CAM used was not harmful to the mother or fetus, the provider’s positive or neutral responses to CAM use can be viewed as beneficial to the provider-patient relationship. They may increase the patient’s willingness to share other information and put the patient more at ease with the provider.

Although obstetrical providers were largely receptive to disclosures of CAM use, they only recommended CAM therapies 15% of the time, while family and friends recommended them almost three-quarters of the time. This dynamic was echoed in studies which found that many people relied on advice from non-healthcare providers, such as friends and relatives, when deciding to use CAM [7,25]. In our patient population, we also looked at whether CAM therapies were more likely to be recommended to patients who had a midwife on their obstetrical team, but the relationship was not statistically significant.

Although the participants perceived little trouble with the safety of the CAM they used, this confidence in CAM safety may be misguided. No harmful effects have been linked to mind-body CAM interventions, such as yoga, hypnotherapy, and imagery [9]. However, the effects of herbal preparations during pregnancy have not been well measured and have been linked to adverse events [10]. In our population, we found that supplement use was more common in the WBN than in the NICU. It is possible the use of this type of CAM does not lead to an increased likelihood that the infant will need admission to the NICU. However, our study was not powered to look at that difference. Since the exact risks associated with CAM use are often unknown, especially in the cases of some Alternative Medicine practices, Biologic-based therapies, and Manipulative and Body-based methods, it is prudent for women to use CAM only if the “benefit is clearly greater than the potential fetal risk” [26].

Weighing benefit with fetal risk may not just be useful when making decisions about CAM use, but also for making decisions about the safety of other medications with little safety data during pregnancy [10]. With around 80% of participants using OTC medication and over half using prescription medication, it is important that the safety and efficacy of these medications is established and that pregnant women are educated about possible risks.

Some limitations to this study include the cross-sectional design, exclusion criteria, sample population, small sample size, and demographic differences between the 2006 and 2013 populations. We excluded post-partum women whose infants died within 24 hours of delivery, women with limited English proficiency (LEP), and women under the age of 18. This may have altered our results, especially in the case of women with LEP, who may have had high levels of cultural CAM use. Because this was a single, large Mid-Atlantic center, these findings may only be generalizable to areas of the United States with demographics similar to this region. Finally, using a larger sample size could have allowed more detailed analysis of relationships between CAM use in the NICU and WBN and between people using specific types of CAM therapies. Future research could address these limitations by doing a multicenter evaluation with better matched groups and analyzing the data using sub-group stratification by different maternal attitudes and demographic characteristics. Future studies should also be done to investigate the safety and efficacy of CAM during pregnancy, the effects of communication about CAM on the provider-patient relationship, the possible relationship between pregnancy complications and when CAM use is initiated, and the increase in dissatisfaction with conventional medicine found between 2006 and 2013.

## Conclusions

Complementary and Alternative Medicine is commonly used by pregnant women in a large Mid-Atlantic center. Its use is not an indictment of conventional medicine; instead, it is often an expression of a woman’s desire to be an active participant in her health. Obstetrical providers are having more discussions about CAM use in 2013 than they were in 2006, but they still do not know about 50% of their patients’ CAM use.

There is a need for increased awareness of CAM use in respect to its frequency, the types of therapies used, and its benefits and risks. Because there is no effective way to use demographics to pinpoint which patients use CAM, providers instead should consider integrating questions about CAM use into their routine patient assessments.

## Competing interests

The authors declare that they have no competing interests.

## Authors’ contributions

RL, AM, UG and DP designed the study. LS and AM were responsible for obtaining informed consent and collecting the surveys. LS and RL analyzed the data. LS drafted the manuscript. All authors edited and approved the final manuscript.

## Pre-publication history

The pre-publication history for this paper can be accessed here:

http://www.biomedcentral.com/1472-6882/14/85/prepub
